# Infectious Cases in General Hospitals

**Published:** 1899-08-26

**Authors:** 


### INFECTIOUS CASES IN GENERAL HOSPITALS.

Dr. Snell, medical officer of health for Coventry, recently read a paper before the Society of Medical Officers of Health on the admission of infectious cases into general hospitals, in which he discussed the questions, What are the diseases for which we can properly advise our authorities to build isolation accommodation; and what are the diseases which it is either unnecessary to isolate, or which can be efficiently dealt with either at home or in a general hospital? It appears, he says, to be indubitable that small-pox is a disease which it is to the interest of the community to isolate rigorously. Typhus fever he would not make any special provision for on account of its rarity. As to scarlet fever, he says it is the general practice to attempt to isolate it in special hospitals, and with this course he agrees; but he points out that this "attempt" at isolation has hitherto been of a very half-hearted character, the proportion of cases isolated being only a little over one-half of the cases which occur. Take the country round we should say that even this was a high estimate, and he says "it may be reasonably open to doubt whether such partial isolation is worth the money that is spent on it, or whether any really practical benefit is derived from it." Still, he would continue to provide isolation accommodation for small-pox and scarlet fever. He then goes on to consider the other infectious diseases. Tuberculosis and erysipelas he puts on one side as being of such limited infectivity that they can be properly treated at home or in general hospitals; and measles, whooping-cough, and chicken-pox are left out of consideration because of the triviality of the latter disease and the expense of dealing with the two former. Having by this somewhat easy-going method of exclusion lightened his task, the author of this paper proceeds to examine the necessity of isolating enteric fever and diphtheria, including in the latter membranous croup. As the result of a specific inquiry addressed to all the general hospitals in the country which had 100 or more beds, 71 in all, he received 68 answers.

Taking first enteric fever. In 56 hospitals these cases are admitted; in seven they are only admitted for observation, or under a mistaken diagnosis; and five hospitals do not admit them at all. Thus, as he says, we have a consensus of opinion which is greatly in favour of the admissibility of the disease into general hospitals, and with this we agree. We think, however, that payment for the maintenance of such patients should be made to the hospitals by the sanitary authorities, and perhaps on a more liberal scale than some places; London, for example.

In regard to diphtheria a much larger difference of opinion seems to prevail. Taking first the cases of membranous croup. Of the 68 hospitals from which replies were received 16 admit cases of membranous croup unreservedly; 46 others admit such cases if urgent, or when they require or are likely to require tracheotomy; while the remaining six hospitals would deal exceptionally with urgent cases as follows: No. 1 would in an urgent case do tracheotomy, and then send the patient a quarter of a mile away to the fever hospital; No. 2 has only had one urgent case in four years; No. 3 has a fever hospital in the same grounds, and such a case would be immediately transferred; No. 4 has a rule passed by the board of management which would necessitate an urgent case being sent away unrelieved; No. 5 would send an urgent case away; No. 6 has, by the board, forbidden the admission of membranous croup, and, according to the house surgeon, "urgent cases are sent home to die. This actually occurred a year ago." *En passant*, we may remark that the scandalous state of affairs shown in these latter cases in all probability results from the very imperfect provision of isolation wards made at some general hospitals, and even at some of those quite recently erected, for the proper separation of cases which are either infectious or are considered to be injurious to other patients in the general wards. In answer to the inquiry whether the admission of cases of croup had led to the spread of the disease to other patients very little was said, and no evidence was given that such had ever been the case. The author of the paper regards these replies as proving conclusively that the possibility of the spread of membranous croup to other patients in a general hospital has been much exaggerated. In regard to ordinary diphtheria, he says that the attempt which has been made in London to isolate this disease has not had an appreciable effect in lessening its prevalence, and from returns made from 20 large provincial towns, comprising towns with populations varying from 50,000 to 100,000, and including, in addition, Manchester, Birmingham, Bradford, and Leicester, he finds that in 10 of these towns an effort is made to isolate diphtheria in the infectious hospital, while in 10 others no such attempt is made. In some few general hospitals ordinary diphtheria is admitted, but this is not the case in most. He adds that he has "seen diphtheria treated in the general wards of a general hospital with no untoward result; but that most of us would probably prefer that such cases should be treated in a side ward," and says that "this side ward need not be an isolated building." But he does not in as many words commit himself to the opinion that ordinary diphtheria should be admitted to general hospitals. So the question, so far as diphtheria is concerned, remains unanswered.

As to membranous croup and enteric fever, his conclusion is that in any town or district where only a limited number of cases are met with they may more advantageously and with greater safety be treated in a general hospital than in a scarlet fever hospital. With this last conclusion we can quite readily concur. But the cases of laryngeal diphtheria, in any epidemic, form but a small proportion of the total number of cases of diphtheria which occur, and we quite fail to see that, even if the general hospitals showed themselves willing to take in cases of laryngeal diphtheria, as they might well do on the ground that they urgently require, or may require, surgical intervention, this would in any way relieve the sanitary authority from the necessity of providing an isolation hospital for the other cases. It would be a large assumption to take for granted that the "duty" of a hospital "to the community" would be held by the governors of any voluntary hospital to include the reception of any such cases as might be sent to it by the sanitary authority.

The prevailing tendency among medical officers of health is to trust very largely to isolation in the management and control of infectious diseases, and if they are to act up to their principles it must very often happen that it is for isolation rather than for treatment that removal from home is demanded. To the hospital manager, however, such a case does not appeal, and he would no more consent to receive a patient who did not require hospital treatment, merely for the sake of isolating it, than he would to take in an old woman who merely wanted food and rest. Moreover, there would be an inherent absurdity in forcibly removing a patient on the plea of the case being one of a dangerous infectious disease, and then placing him in a hospital full of sick and susceptible patients. Compulsory removal must go by the board at once, unless such removal is to eventuate in isolation. In any case, the question of the admission of diphtheria is an important one, and one on which hospital managers should endeavour to arrive at some definite conclusion. There is a good deal of evidence to show that in the case of many of the diseases which are properly enough regarded as "dangerous" and "infectious," the infection is far less likely to be disseminated amidst good hospital surroundings than in the houses where it has originated. But to ensure such a degree of immunity a hospital must be both built and managed on much more expensive principles than suffice for the ordinary run of non-infectious ailments; the floor space, the cubic space, and the nursing staff must all be arranged on a more elaborate scale than in ordinary hospitals; and although it may be true that "a case" of diphtheria taken into a well-managed hospital may not do any harm, there is very little evidence to show that a group of cases can safely be admitted without danger. If we are to look upon the very elaborate precautions taken in the infectious hospitals to isolate diphtheria as in any way justified, we cannot admit that it would be right in an ordinary hospital, in which such precautions are out of the question, to accept as an ordinary thing the diphtheria cases sent in by the sanitary authority, for it must be remembered that they are pretty sure to be sent in not singly but in groups, as they are generally met with. Still every general hospital should be prepared for all emergencies, and should be provided with such an amount of isolation accommodation as to be able to deal satisfactorily with such individual urgent cases as may arise in ordinary course, and especially those of laryngeal diphtheria, which urgently require operative treatment. Isolation is another matter; it is a thing done not for the benefit of the individual but for the public good, and must be undertaken and paid for by the public authority.

